# APC orchestrates microtubule dynamics by acting as a positive regulator of KIF2A and a negative regulator of CLASPs

**DOI:** 10.1016/j.cellin.2024.100210

**Published:** 2024-10-11

**Authors:** Yong Wang, Xinping Liu, Zheng Liu, Shasha Hua, Kai Jiang

**Affiliations:** a State Key Laboratory of Oral & Maxillofacial Reconstruction and Regeneration, Key Laboratory of Oral Biomedicine Ministry of Education, Hubei Key Laboratory of Stomatology, School & Hospital of Stomatology, Medical Research Institute, Wuhan University, Wuhan, 430071, China; b Frontier Science Center for Immunology and Metabolism, Wuhan University, Wuhan, 430071, China; c The Institute for Advanced Studies, TaiKang Center for Life and Medical Sciences, Hubei Key Laboratory of Cell Homeostasis, College of Life Sciences, Wuhan University, Wuhan, 430072, China

**Keywords:** Microtubule, Adenomatous polyposis coli protein, APC, CLASPs, KIF2A

## Abstract

Tumor suppressor protein Adenomatous polyposis coli protein (APC) is an EB-binding and microtubule (MT) plus end-tracking protein; however, how exactly APC regulates MT dynamics remains elusive. Here, we show that in LLC-PK1 cells, APC and KIF2A, an MT depolymerase, form a complex clustering at the cell edge and destabilize MTs at the MT plus ends. Further biochemical characterization and mutational analysis reveal key residues for the APC-KIF2A interaction. In addition, APC counteracts the major MT-stabilizer CLASPs at MT plus ends and promotes directional cell migration via modulating cell adhesion force. Reconstitution experiments demonstrate that APC potentiates KIF2A-induced MT catastrophes and antagonizes the stabilizing effect of CLASP2 *in vitro*. In summary, APC functions as a positive regulator of MT-destabilizer and a negative regulator of MT-stabilizer to orchestrate MT dynamics.

## Highlights

• APC and KIF2A form a complex clustering at the cell edge and destabilize MTs at the MT plus ends.
• APC counteracts MT-stabilizer CLASPs at MT plus ends and regulates adhesion forces and cell migration.
• APC potentiates KIF2A-induced MT catastrophes and antagonizes the stabilizing effect of CLASP2 in vitro.

## Graphical abstract

**Figure:**
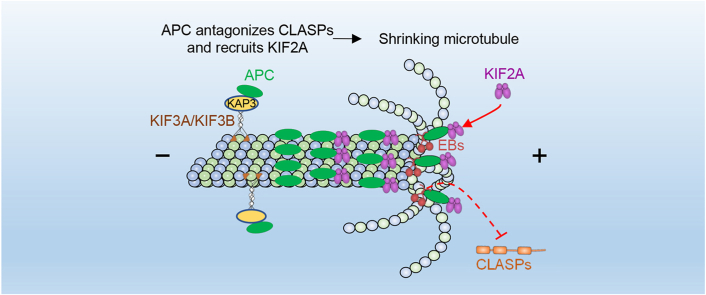

## 1 Introduction

Microtubules (MTs) are essential for intracellular transport, cell migration, cell polarity, and cell division. MTs are intrinsic polarized dynamic polymers assembled from asymmetric αβ-tubulin heterodimers head-to-tail. The β-tubulin-exposed plus ends are more dynamic than the α-tubulin-exposed minus ends (Akhmanova & Kapitein, 2022; Desai & Mitchison, 1997).

The MT plus ends are the primary sites for the modulation of MT dynamics. A large number of proteins, called MT plus-end tracking proteins (+TIPs), associate growing MT plus ends and regulate plus-end dynamics and their interaction with other cellular structures (Akhmanova & Steinmetz, 2015). The master regulators of the +TIP complexes are the End Binding proteins (EBs) that autonomously track the polymerizing ends by recognizing an intermediate nucleotide state of MTs (Bieling et al., 2007; Maurer et al., 2012; Zhang et al., 2015). Many other + TIPs, such as CLASPs, KIF2C/MCAK, and CLIP-170 can be recruited by EBs to plus-ends via their SxIP motifs or CAP-Gly domains (Akhmanova & Steinmetz, 2015; Honnappa et al., 2009; Jiang et al., 2012; Weisbrich et al., 2007).

Tumor suppressor protein Adenomatous polyposis coli protein (APC) is the prototype of EB-binding and SxIP motif-containing protein (Honnappa et al., 2009; Su et al., 1995). APC has been suggested to stabilize MTs in several cell lines and neurons and in bulk *in vitro* assays (Kroboth et al., 2007; Lavrsen et al., 2023; Marshall et al., 2011; Nakamura et al., 2001; Reilein & Nelson, 2005; Togo, 2009; Wen et al., 2004; Yokota et al., 2009; Zumbrunn et al., 2001). However, two recent *in vitro* studies showed contradictory results regarding the effect of APC on MT dynamics. One reported that APC has no impact on MT dynamics *in vitro* (Baumann et al., 2022), while the other reported that APC stabilizes MTs by promoting rescues (Serre et al., 2023). To make things more complicated, MT looping or curling has been observed after the knockout or knockdown of APC in neurons (Chen, Tian, et al., 2011; Eom et al., 2011; Purro et al., 2008). Considering that curvy MTs are long-lived stable MTs and are often marked by acetylation (Janke & Montagnac, 2017; Piperno et al., 1987), it is tempting to speculate that APC might play a role in establishing a more dynamic rather than stable MT network. This is supported by the finding that APC2, the homolog of APC, promotes MT dynamics in dendrites (Kahn et al., 2018). Thus, it remains elusive exactly how APC regulates MT dynamics alone or together with other binding partners.

Besides localizing at the growing MT ends, APC can also be transported by hetero-trimeric Kinesin-2 complex (KIF3A/3B/KAP3) through the APC-KAP3 interaction and hence clusters at the cell edge (Baumann et al., 2020; Jimbo et al., 2002). CLASPs, another + TIP family, concentrate at the cell margin and stabilize the cortical MTs by promoting MT rescues and inhibiting catastrophes (Aher et al., 2018; Akhmanova et al., 2001; Lawrence et al., 2018; Majumdar et al., 2018; Yu et al., 2016). Whether these two cell edge-enriched + TIPs are functionally connected has not been investigated.

Here, our biochemical data demonstrate that APC interacts with the MT depolymerase KIF2A, and further mutational analysis reveals key residues for this binding. Our cellular experiments show that the APC-KIF2A complex clusters at the cell periphery and destabilizes MTs at the MT plus ends. The localization of KIF2A depends on APC. Moreover, APC counteracts the major MT-stabilizer CLASPs at MT plus ends and regulates adhesion forces and cell migration. Mechanistically, *in vitro* reconstitution assays reveal that APC promotes KIF2A-induced MT catastrophes and outcompetes the stabilizing effect of CLASP2. We propose that APC regulates MT dynamics by acting as both a positive regulator of MT-destabilizer and a negative regulator of MT-stabilizer.

## 2 Results

### 2.1 APC and KIF2A form a complex clustering at the cell edge and function in destabilizing MTs at the MT plus ends

To gain insight into the function of APC, we attempted to identify its binding partners. Extracts of HEK293T cells expressing Bio-GFP-APC together with the biotin ligase BirA were used for streptavidin pull-down assays, and the resulting proteins were analyzed by mass spectrometry. In addition to the known proteins that are in complex with APC, the MT depolymerase KIF2A was also present in the APC pull-down (Figs. S1A and B).

**Fig. 1:**
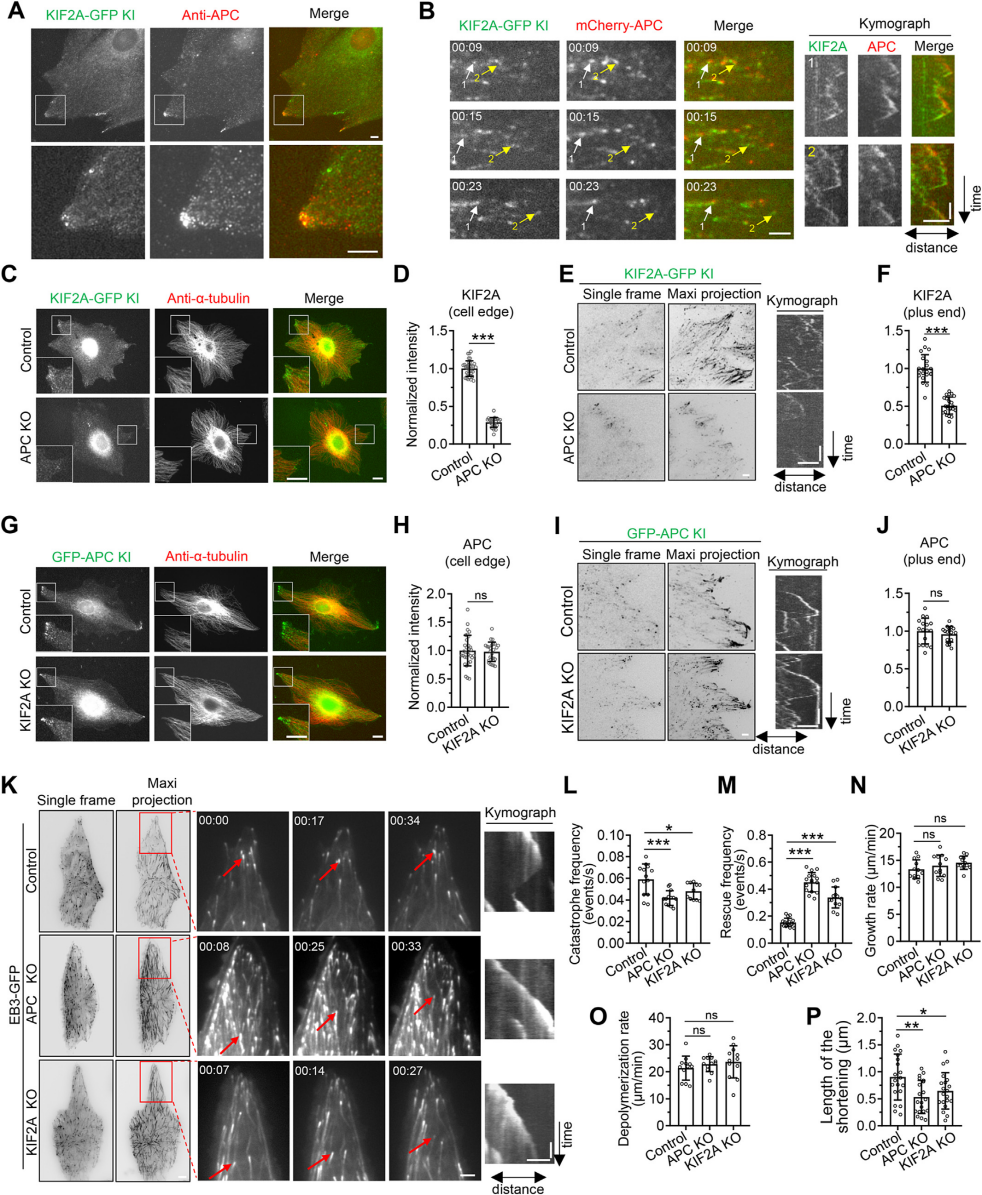
**APC and KIF2A form a complex clustering at the cell edge and function in destabilizing MTs at the MT plus ends** (A) Immunofluorescence staining of APC in KIF2A-GFP-Strep knock-in LLC-PK1 cells. Lower panels show enlargements of boxed areas in the upper panels. (B) TIRF microscopy time-lapse images and kymograph showing KIF2A and APC tracking the MT plus-ends in KIF2A-GFP-Strep knock-in LLC-PK1 cell line transiently transfected with mCherry-APC. Arrows indicate representative MTs. Scale bars: horizontal, 2 μm; vertical, 20 s (C) Immunofluorescence staining of α-tubulin in control KIF2A-GFP knock-in LLC-PK1 cells or APC KO/KIF2A-GFP-Strep knock-in double-engineered LLC-PK1 cell line. Insets show enlargements of boxed areas. (D) Quantification of intensities of KIF2A at the cell edge for the experiments shown in (C). *n* = 27–35 cells from three independent experiments. (E) TIRF live-cell imaging of control KIF2A-GFP-Strep knock-in LLC-PK1 cells or APC KO/KIF2A-GFP-Strep knock-in double-engineered LLC-PK1 cell line. The left panels show the single frame and the maximum intensity projection over 200 frames (100 s) of the GFP channel. The right panels show representative kymographs illustrating the behaviors of KIF2A at MT plus-end in control or APC KO cells. Scale bars: horizontal, 2 μm; vertical, 20 s (F) Quantification of intensities of KIF2A at the MT plus-end for the experiments shown in (E). *n* = 20–23 cells from three independent experiments. (G) Immunofluorescence staining of α-tubulin in control Strep-GFP-APC knock-in LLC-PK1 cells or KIF2A KO/Strep-GFP-APC knock-in double-engineered LLC-PK1 cell line. Insets show enlargements of boxed areas. (H) Quantification of intensities of APC at the cell edge for the experiments shown in (G). n.s, no significance in unpaired two-tailed *t*-test. *n* = 25–32 cells from three independent experiments. (I) TIRF live-cell imaging of control Strep-GFP-APC knock-in LLC-PK1 cells or KIF2A KO/Strep-GFP-APC knock-in double-engineered LLC-PK1 cell line. The left panels show the single frame and the maximum intensity projection over 200 frames (100 s) of the GFP channel. The right panels show representative kymographs illustrating the behaviors of APC at MT plus-end in control or KIF2A KO cells. Scale bars: horizontal, 2 μm; vertical, 20 s (J) Quantification of intensities of APC at the MT plus-end for the experiments shown in (I). n.s, no significance in unpaired two-tailed *t*-test. *n* = 13–20 cells from two independent experiments. (K) TIRF live-cell imaging of control EB3-GFP knock-in LLC-PK1 cell line, APC KO/EB3-GFP knock-in double-engineered LLC-PK1 cell line, and KIF2A KO/EB3-GFP knock-in double-engineered LLC-PK1 cell line. The left panels show the single frame and the maximum intensity projection over 200 frames (100 s) of the GFP channel. Middle panels show time-lapse images of boxed areas. The right panels show corresponding kymographs of MTs indicated by arrows in the middle panels. Scale bars: horizontal, 2 μm; vertical, 20 s (L–P) Quantification of the catastrophe frequency (L), rescue frequency (M), growth rate (N), depolymerization rate (O), and the length of the shortening excursions (P) of MT plus-ends in the vicinity of the cell edge for experiments shown in (K). n.s, no significance in unpaired two-tailed *t*-test. *n* = 11–20 cells from three independent experiments. Data information: Unless otherwise stated, data represent mean ± SD. ∗*p* < 0.05; ∗∗*p* < 0.01; ∗∗∗*p* < 0.001; two-tailed *t*-test (unpaired). Scale bars, 5 μm.

Immunostaining in three cell lines using an anti-APC antibody revealed that APC showed prominent accumulation in punctate clusters in extending membranes in pig kidney-derived LLC-PK1 cells, which is consistent with previous reports (Abbott & Nathke, 2023; Nathke et al., 1996), whereas such localization was weak in HeLa and U2OS cells (Figs. S1C and D). Therefore, LLC-PK1 cells were chosen for the current study. We then generated the Strep-GFP-APC and KIF2A-GFP-Strep knock-in LLC-PK1 cell line via CRISPR/Cas9 technology (Figs. S1E–J). Immunofluorescent staining in KIF2A-GFP-Strep knock-in cell line revealed that endogenous KIF2A and APC formed overlapping clusters at the cell edge (Fig. 1A). Further live cell imaging showed that APC could often track both polymerizing and depolymerizing MT plus ends together with KIF2A (Fig. 1B). Knockout of APC in LLC-PK1 cells dramatically reduced the localization of KIF2A at MT plus ends and its clustering at the cell edge, whereas knockout of KIF2A had no impact on the localization of APC, suggesting that KIF2A recruitment depends on APC (Fig. 1C–J, Figs. S1K–O).

To understand how APC and KIF2A affect MT dynamics, we performed live cell imaging in control and in APC or KIF2A knockout LLC-PK1 cell lines stably expressing EB3-GFP. Knockout of APC and KIF2A had no significant impact on the growth rate and depolymerization rate but dramatically reduced the catastrophe frequency and length of the shortening excursions, and increased rescue frequency, with the effect of APC knockout being more pronounced (Fig. 1K–P). These results suggest that beyond the recruitment of MT depolymerase KIF2A, APC plays a significant role in destabilizing MTs via some other mechanisms.

### 2.2 APC counteracts MT-stabilizer CLASPs at MT plus ends and regulates adhesion forces and cell migration

**Fig. 2:**
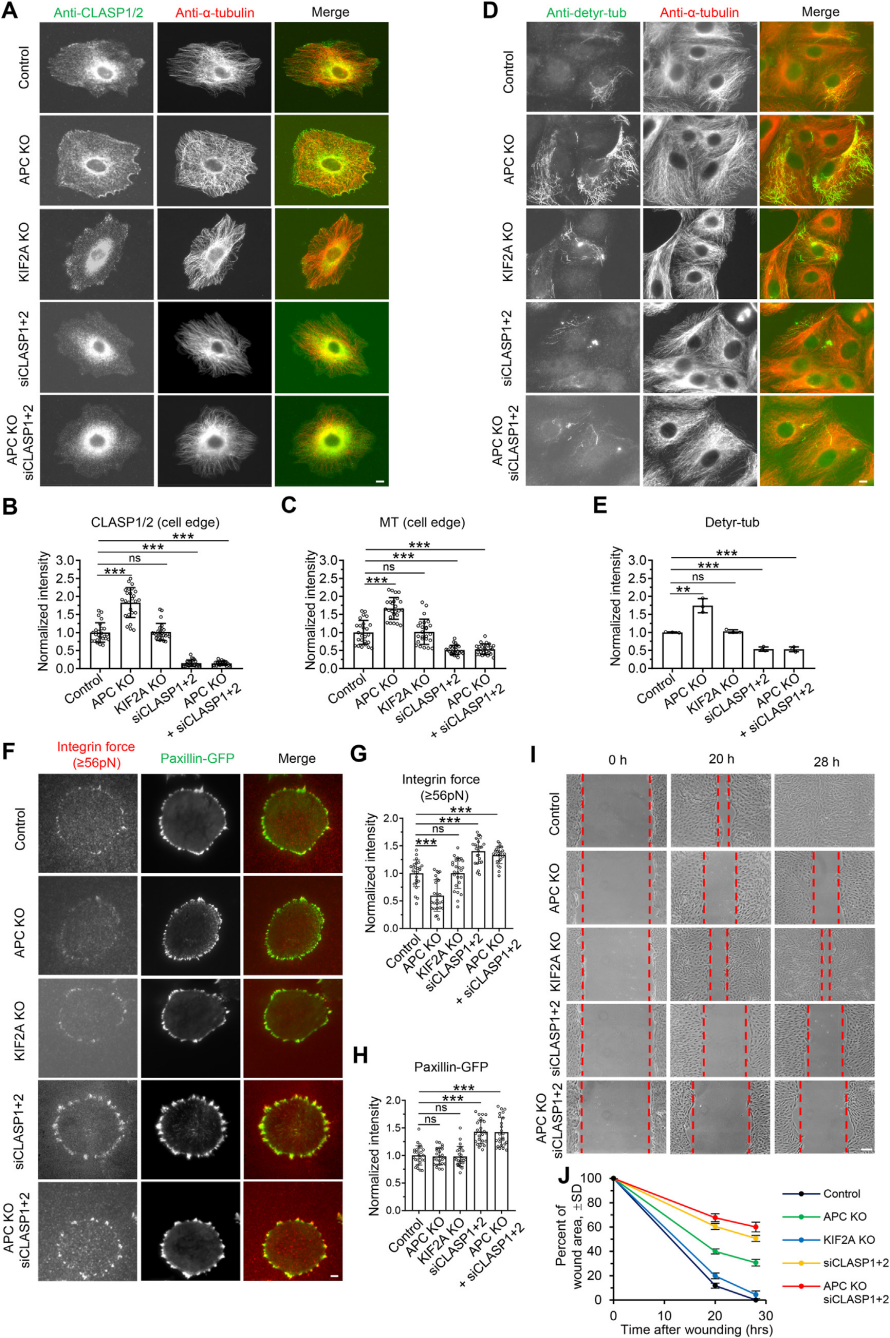
**APC counteracts MT-stabilizer CLASPs at MT plus ends and regulates adhesion forces and cell migration** (A) Immunofluorescence staining of CLASP1/2 (green) and α-tubulin (red) in control, indicated KO LLC-PK1 cell lines, CLASP1/2 depleted-LLC-PK1 cells, or CLASP1/2- depleted APC KO LLC-PK1 cells. (B–C) Quantification of intensities of CLASP1/2 (B) and MT (C) at the cell edge for the experiments shown in (A). *n* = 25 cells from three independent experiments. (D) Immunofluorescence staining of detyrosinated-tubulin (green) and α-tubulin (red) in control, indicated KO LLC-PK1 cell lines, CLASP1/2-depleted LLC-PK1 cells, or CLASP1/2-depleted APC KO LLC-PK1 cells. (E) Quantification of intensities of detyrosinated-tubulin for the experiments shown in (D). *n* = 3 independent experiments (25–36 cells). (F–H) Representative TIRF microscopy images of cells expressing paxillin-GFP incubated on coverslips coated with 56 pN DNA probe (F) and corresponding quantification of tension signal intensity (G) and paxillin intensity (H). Scale bars, 2 μm. (I–J) Monolayer wound-healing assays in control, indicated KO LLC-PK1 cell lines, CLASP1/2-depleted LLC-PK1 cells, or CLASP1/2-depleted APC KO LLC-PK1 cells. Phase-contrast images at the indicated time points are shown in (I). Quantification was based on three independent experiments (J). Data information: Unless otherwise stated, data represent mean ± SD. ∗∗*p* < 0.01; ∗∗∗*p* < 0.001; two-tailed *t*-test (unpaired). Scale bars, 5 μm.

To explore other possible mechanisms, we investigated whether there was a relationship between APC, KIF2A, and the major MT stabilizer CLASPs. Immunofluorescent staining in the GFP-APC knock-in LLC-PK1 cell line with antibodies against CLASP1/2 and paxillin, a marker for focal adhesion, revealed that APC colocalizes with a subpopulation of CLASP1/2, which is distributed broadly at the cell edge and often accumulated in the vicinity of focal adhesions (Figs. S2A–C). Knockout of APC increased the intensities of CLASPs and MTs at the cell edge by ∼83% and ∼67%, respectively (Fig. 2A–C). Consistently, the total mass of detyrosinated MT, a marker for stable MTs, was also dramatically increased in APC knockout cells (Fig. 2D and E). However, the knockout of KIF2A had little impact on the distribution of CLASPs, the MT density at the cell edge, and the intensity of detyrosinated MTs (Fig. 2A–E). These results suggest that APC, but not KIF2A, can counteract CLASPs at the MT plus ends at the cell periphery, thereby providing another mechanism to destabilize MTs. Supporting this, depleting CLASPs in APC knockout cells led to a significant reduction in the MT density at the cell edge and the intensity of detyrosinated MTs, compared to control cells. The density and intensity were similar to cells lacking CLASPs alone (Fig. 2A–E).

It has been indicated that stable MTs can function as tracks for modulating the dynamics of focal adhesion (Kaverina et al., 1998, 1999; Stehbens et al., 2014). APC can also directly affect focal adhesion dynamics through its actin-binding and nucleation activities (Juanes et al., 2017, 2019). However, whether APC would influence the adhesion force is unknown. To address this question, we used a recently developed DNA probe RSDTP 56 pN for force measurement (Li et al., 2021). The intensity of RSDTP 56 pN was reduced by ∼40% in APC knockout cells but not in KIF2A knockout cells, while the intensity of paxillin was unchanged, demonstrating that although the absence of APC would not affect the mean size of focal adhesion, it could reduce the adhesion force (Fig. 2F–H). By contrast, depleting CLASPs in LLC-PK1 cells increased the size of focal adhesion and adhesion force, as revealed by the elevated intensities of paxillin and RSDTP 56 pN (Fig. 2F–H), which is consistent with a previous report showing that CLASPs facilitate focal adhesion disassembly by localized exocytosis and extracellular matrix degradation, disrupting integrin-matrix connections (Stehbens et al., 2014). Moreover, we found that depleting CLASPs in APC knockout cells could increase the adhesion force to a level similar to that of depleting CLASPs alone, suggesting that APC regulates the adhesion force by antagonizing the impact of CLASPs (Fig. 2F–H). To investigate the functional consequence further, we performed monolayer wound healing assays. Cells that lack APC, CLASPs, or KF2A showed a reduced ability to close the monolayer. CLASPs depletion had the most significant impact, followed by APC knockout, and then KIF2A knockout, which had the lowest impact (Fig. 2I and J). Depleting CLASPs in APC knockout cells caused a slightly more significant defect in monolayer closure than the absence of CLASPs alone (Fig. 2I and J). Collectively, these results suggest that APC antagonizing CLASPs is beneficial for maintaining the balance of adhesion force and that either insufficient or excessive adhesion force is detrimental to cell migration.

### 2.3 Biochemical characterization and mutational analysis of the APC-KIF2A complex

**Fig. 3:**
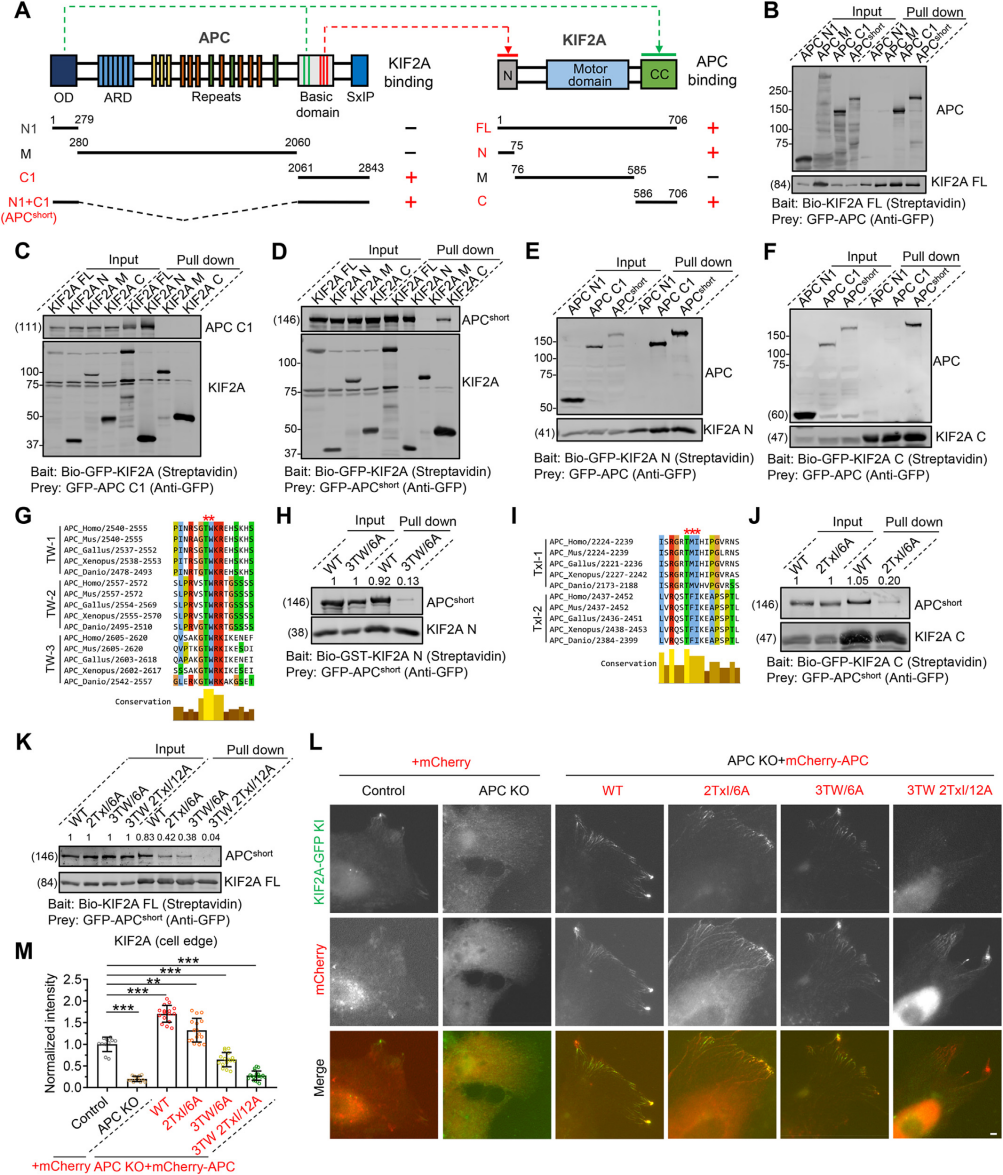
**Biochemical characterization and mutational analysis of the APC-KIF2A complex** (A) Left, schematic overview of the domain organization of APC and the deletion mutants and a summary of their interactions with KIF2A. Right, schematic overview of the domain organization of KIF2A and the deletion mutants and summary of their interactions with APC. OD, oligomerization domain; ARD, Armadillo repeat domain; CC, Coiled-coil. (B) Streptavidin pull-down assays with extracts of HEK293T cells expressing Bio-tagged KIF2A full length (FL) (bait) together with the indicated GFP-tagged APC truncations (prey), analyzed by western blotting with GFP antibody. The Bio-tagged bait proteins were detected by IRDye® 680RD Streptavidin. (C) Streptavidin pull-down assays with extracts of HEK293T cells expressing Bio-GFP-tagged KIF2A FL and the indicated truncations (bait) together with GFP-tagged APC C1 (prey), analyzed by western blotting with GFP antibody. The Bio-GFP-tagged bait proteins were detected by IRDye® 680RD Streptavidin. (D) Streptavidin pull-down assays with extracts of HEK293T cells expressing Bio-GFP-tagged KIF2A FL and the indicated truncations (bait) together with GFP-tagged APC^short^ (prey), analyzed by western blotting with GFP antibody. The Bio-GFP-tagged bait proteins were detected by IRDye® 680RD Streptavidin (E) Streptavidin pull-down assays with extracts of HEK293T cells expressing Bio-GFP-tagged KIF2A N (bait) together with the indicated GFP-tagged APC truncations (prey), analyzed by western blotting with GFP antibody. The Bio-GFP-tagged bait proteins were detected by IRDye® 680RD Streptavidin. (F) Streptavidin pull-down assays with extracts of HEK293T cells expressing Bio-GFP-tagged KIF2A C (bait) together with the indicated GFP-tagged APC truncations (prey), analyzed by western blotting with GFP antibody. The Bio-GFP-tagged bait proteins were detected by IRDye® 680RD Streptavidin. (G) Alignment of KIF2A N-binding region in APC from five vertebrate species. The threonine and tryptophan residues critical for KIF2A N-binding were indicated with asterisks. (H) Streptavidin pull-down assay with extracts of HEK293T cells expressing Bio-GST-tagged KIF2A N (bait) together with GFP-tagged wild-type (WT) APC^short^ or its 3TW/6A mutant (prey), analyzed by western blotting with GFP antibody. The Bio-GST-tagged bait proteins were detected by IRDye® 680RD Streptavidin. (I) Alignment of KIF2A C-binding region in APC from five vertebrate species. The threonine, methionine (or phenylalanine), and isoleucine residues critical for KIF2A C-binding were indicated with asterisks. (J) Streptavidin pull-down assay with extracts of HEK293T cells expressing Bio-GFP-tagged KIF2A C (bait) together with GFP-tagged WT APC^short^ or its 2TxI/6A mutant (prey), analyzed by western blotting with GFP antibody. The Bio-GFP-tagged bait proteins were detected by IRDye® 680RD Streptavidin. (K) Streptavidin pull-down assay with extracts of HEK293T cells expressing Bio-tagged KIF2A FL (bait) together with GFP-tagged WT APC^short^ or its indicated mutants (prey), analyzed by western blotting with GFP antibody. The Bio-tagged bait proteins were detected by IRDye® 680RD Streptavidin. (L) Left, images of control KIF2A-GFP-Strep knock-in LLC-PK1 cell line or APC KO/KIF2A-GFP-Strep knock-in double-engineered LLC-PK1 cell line transiently transfected with control mCherry vector. Right, images of APC KO/KIF2A-GFP-Strep knock-in double-engineered LLC-PK1 cell line transiently transfected with mCherry-tagged WT APC or its indicated mutants. (M) Quantification of intensities of KIF2A at the cell edge for the experiments shown in (L). *n* = 13–20 cells from three independent experiments. Data information: Data represent mean ± SD. ∗∗*p* < 0.01; ∗∗∗*p* < 0.001; two-tailed *t*-test (unpaired). Scale bars, 5 μm.

Next, we explored the interaction between APC and KIF2A in more detail. APC contains an oligomerization domain at its N-terminus (APC-N1), an Armadillo repeat domain (ARD) and 15-aa, 20-aa, SAMP repeats in the middle (APC-M), and a basic domain and an EB-binding domain with multiple SxIP EB-binding motifs at its C-terminus (APC-C1) (Fig. 3A and Fig. S3A). KIF2A encompasses an N-terminal globular domain (KIF2A-N), a motor domain in the middle (KIF2A-M), and a C-terminal coiled-coil domain (KIF2A-C) (Fig. 3A). Our first round of Streptavidin pull-down assay showed that APC's fragment C1 is the minimal region required for KIF2A binding (Fig. 3B). Moreover, we found that APC C1 could bind KIF2A-N (Fig. 3C and E), while a chimeric fragment (APC-N1+C1, called APC^short^ hereafter) could also bind KIF2A-C (Fig. 3D and F), suggesting that the affinity of APC C1 alone is sufficient for its binding to KIF2A-N but insufficient for KIF2A-C, while the presence of an oligomerization domain could enhance its affinity for KIF2A-C. We then generated additional deletion constructs to narrow down the interaction site further and found that three and two conserved linear repeats located within APC C1 were necessary for interacting with KIF2A-N and KIF2A-C, respectively (Figs. S3A–I). Substitution of the conserved “TW” and “TxI” by alanine in the context of either single linear repeats or APC^short^ could dramatically reduce their interaction with KIF2A-N and KIF2A-C, respectively (Fig. 3G–J and Fig. S3J and K). Moreover, when all three “TW” and two “TxI” were mutated simultaneously in APC^short^, its binding affinity for KIF2A-FL (full length) was almost abolished (Fig. 3K).

To examine the effects of these mutations under physiological conditions, we introduced WT and mutated versions of mCherry-APC into KIF2A-GFP KI/APC KO cells. Expression of WT mCherry-APC not only fully rescued the impaired localization of KIF2A caused by APC KO but also, in fact, enhanced its clustering at the cell edge, most likely due to an average 3.6-fold higher exogenous APC expression level than the endogenous level of control cells and the resulting stronger recruitment effect (Fig. 3L and M and Fig. S3L). Compared to WT mCherry-APC, the ability to rescue KIF2A localization was reduced in cells expressing mCherry-APC harboring mutations of three “TW” (3TW/6A) or two “TxI” (2TxI/6A), with 3TW/6A being more evident. Strikingly, mCherry-APC harboring simultaneous mutations of all three “TW” and two “TxI” (3TW 2TxI/12A) almost completely lost the rescue ability (Fig. 3L and M). These data further validate the importance of these residues and demonstrate that the targeting of KIF2A to the MT plus-end and cell edge can be achieved by direct binding to APC.

Reciprocally, we also examined which residues of KIF2A facilitated the interaction with APC. AlphaFold predicts the N-terminal globular domain of KIF2A to be a barrel-like structure (Fig. S4A). Structure similarity search against the PDB database by Foldseek reveals that the folding of KIF2A (1–76 aa) is similar to that of the Tudor domains recognizing methylated lysine or arginine residues (Fig. S4B) (Chen, Nott, et al., 2011; Lu & Wang, 2013). Our mutational analysis revealed that residues on top of the barrel (marked in red in Fig. S4A) but not at its side (marked in grey) are critical for APC binding, with Arg19 being of particular importance (Fig. S4C). Attempts to identify critical residues on the coiled-coil domain of KIF2A were unsuccessful. However, unexpectedly, we found that the mutations in the KIF2A motor domain, which diminish its depolymerase activity, and have been linked to a variety of neurodevelopmental and neurological disorders (Poirier et al., 2013; Ruiz-Reig et al., 2024), led to the formation of MT bundles and further the recruitment of endogenous APC to these bundles, suggesting that the altered APC localization may also contribute to KIF2A-related pathogenesis (Fig. S4D).

Collectively, we conclude that APC interacts with KIF2A, and the interface responsible for this interaction consists of the conserved linear motifs at the basic domain of APC and the N-terminal globular domain and C-terminal coiled-coil domain of KIF2A.

### 2.4 APC potentiates KIF2A-mediated MT catastrophe *in vitro*

**Fig. 4:**
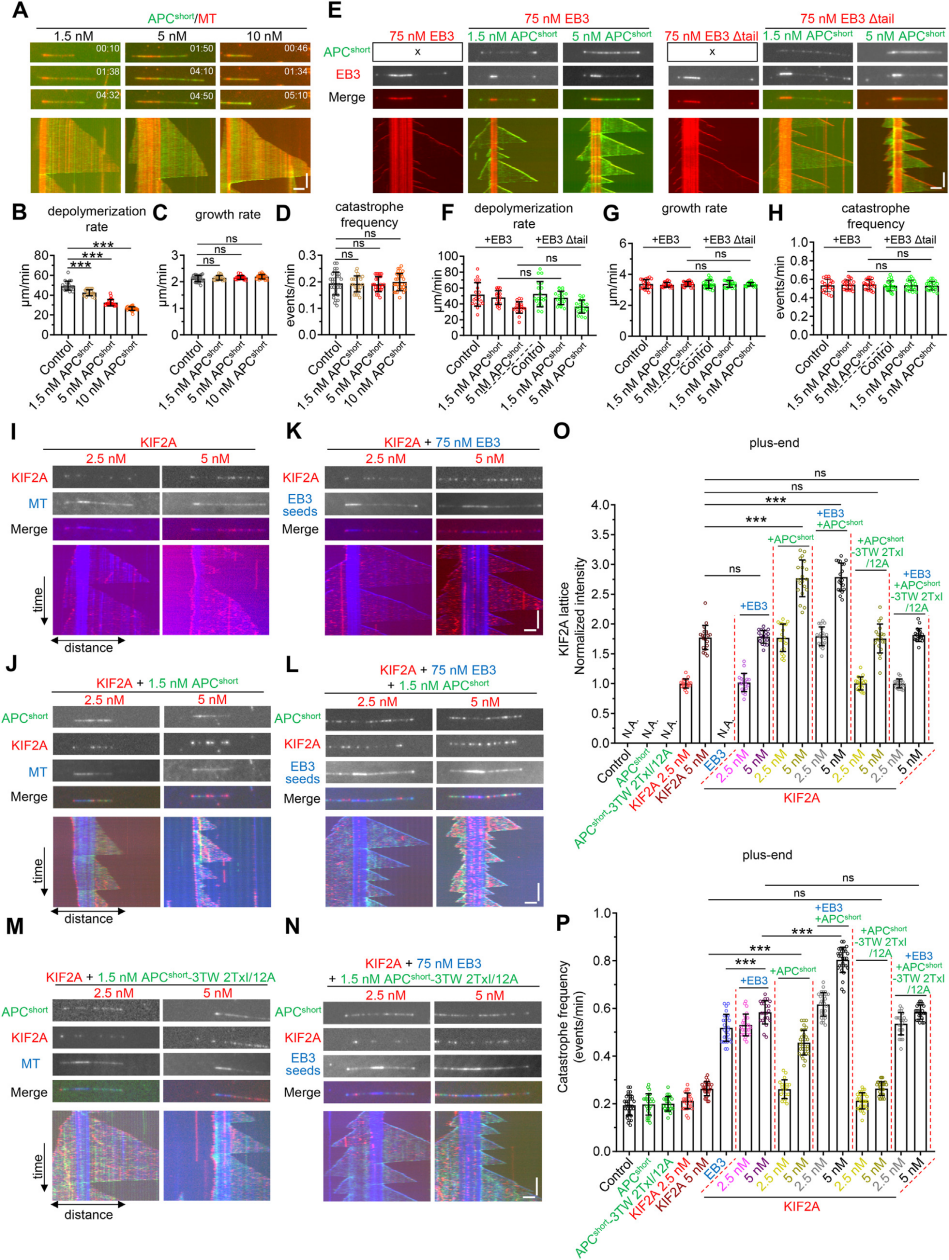
**APC potentiates KIF2A-mediated MT catastrophes *in vitro*** (A) Images and corresponding kymographs showing the behavior of GFP-APC^short^ on dynamic MTs at different concentrations. (B–D) Quantification of MT plus-end depolymerization rate (B), growth rate (C), and catastrophe frequency (D) in the presence of GFP-APC^short^ at indicated concentrations. *n* = 21–31 MTs from three independent experiments. (E) Images and corresponding kymographs of dynamic MTs grown in the presence of 75 nM mCherry-EB3 or mCherry-EB3 delta Tail alone or together with GFP-APC^short^ at indicated concentrations. (F–H) Quantification of MT plus-end depolymerization rate (F), growth rate (G), and catastrophe frequency (H) for the experiments shown in (E). *n* = 20–23 MTs from 2 to 3 independent experiments. (I) Images and corresponding kymographs of dynamic MTs (shown here in blue) grown in the presence of KIF2A-SNAP labeled with Alexa Fluor 647 (shown here in red) at indicated concentrations. (J) Images and corresponding kymographs of dynamic MTs grown in the presence of WT GFP-APC^short^ and KIF2A-SNAP at indicated concentrations. (K) Images and corresponding kymographs of dynamic MTs grown in the presence of mCherry-EB3 (shown here in blue) and KIF2A-SNAP (shown here in red) at indicated concentrations. (L) Images and corresponding kymographs of dynamic MTs grown in the presence of WT GFP-APC^short^, KIF2A-SNAP (shown here in red), and mCherry-EB3 (shown here in blue) at indicated concentrations. (M) Images and corresponding kymographs of dynamic MTs grown in the presence of GFP-APC^short^ mutant and KIF2A-SNAP (shown here in red) at indicated concentrations. (N) Images and corresponding kymographs of dynamic MTs grown in the presence of GFP-APC^short^ mutant, KIF2A-SNAP (shown here in red), and mCherry-EB3 (shown here in blue) at indicated concentrations. (O–P) Quantification of KIF2A intensity on MT lattices (O) and catastrophe frequency (P) for the experiments shown in (I–N). *n* = 20–31 MTs from 2 to 3 independent experiments. Data information: Data represent mean ± SD. ∗∗∗*p* < 0.001; two-tailed *t*-test (unpaired). Scale bars: horizontal, 2 μm; vertical, 2 min.

To gain further insight into how APC and KIF2A work together, we set out to reconstitute their activities by performing *in vitro* MT dynamics assays using proteins purified from HEK293T cells. Of note is that APC^short^ was used in the subsequent experiments because the large molecular mass of full-length APC made it difficult to obtain the full-length protein. In the presence of tubulin alone, GFP-tagged APC^short^ showed MT lattice binding. When APC^short^ concentration reached 5 nM and above, accumulation of APC^short^ on shrinking but not growing plus ends was also observed (Fig. 4A). APC^short^ slowed down the plus-end depolymerization rate in a dose-dependent manner but had little impact on growth rate and catastrophe frequency (Fig. 4A–D). No rescue events were observed at all tested APC^short^ concentrations. When mCherry-EB3 was included in the assay, APC^short^ was strongly recruited to MT plus-ends at 1.5 nM and 5 nM (Fig. 4E). MT tip recruitment was abolished by the removal of the acidic tail of EB3 (EB3 delta Tail) (Fig. 4E), as these polypeptide sequences are essential for the binding between SxIP Motif-Containing EB binding proteins and EBs (Jiang et al., 2012; Montenegro Gouveia et al., 2010). MT dynamics parameters, including plus-end depolymerization rate, growth rate, and catastrophe frequency, were similar in the presence of EB3 and EB3 delta Tail (Fig. 4F–H), suggesting that no matter whether tip recruitment by EB3 or not, APC had no stabilization or destabilization effect.

We also investigated the behaviors of mCherry-KIF2A alone at 2.5 nM and 5 nM. KIF2A displayed weak MT lattice binding at these two concentrations and could moderately increase the plus-end catastrophe frequency at 5 nM but not 2.5 nM (Fig. 4I,O and P). When added together with 1.5 nM APC^short^, KIF2A intensities along MT lattices were dramatically increased because of the recruitment by APC^short^ (Fig. 4J and O). Moreover, at 5 nM KIF2A, a roughly twofold increase in the catastrophe frequency was observed in the presence of APC^short^ than in its absence (Fig. 4I, J and P). We further introduced EB3, which can promote catastrophe by itself, in the assay. In the background of EB3, the addition of APC^short^ could also promote KIF2A-induced catastrophe, especially for KIF2A at 5 nM (Fig. 4K, L and P). Of note is that the KIF2A-binding deficient APC^short^ mutant failed to do so, no matter in the presence or absence of EB3 (Fig. 4M, N and P), suggesting that the direct interaction between APC and KIF2A is crucial to making this complex a potent MT destabilizer that induces catastrophe.

### 2.5 APC antagonizes the stabilization effect of CLASP2 *in vitro*

**Fig. 5:**
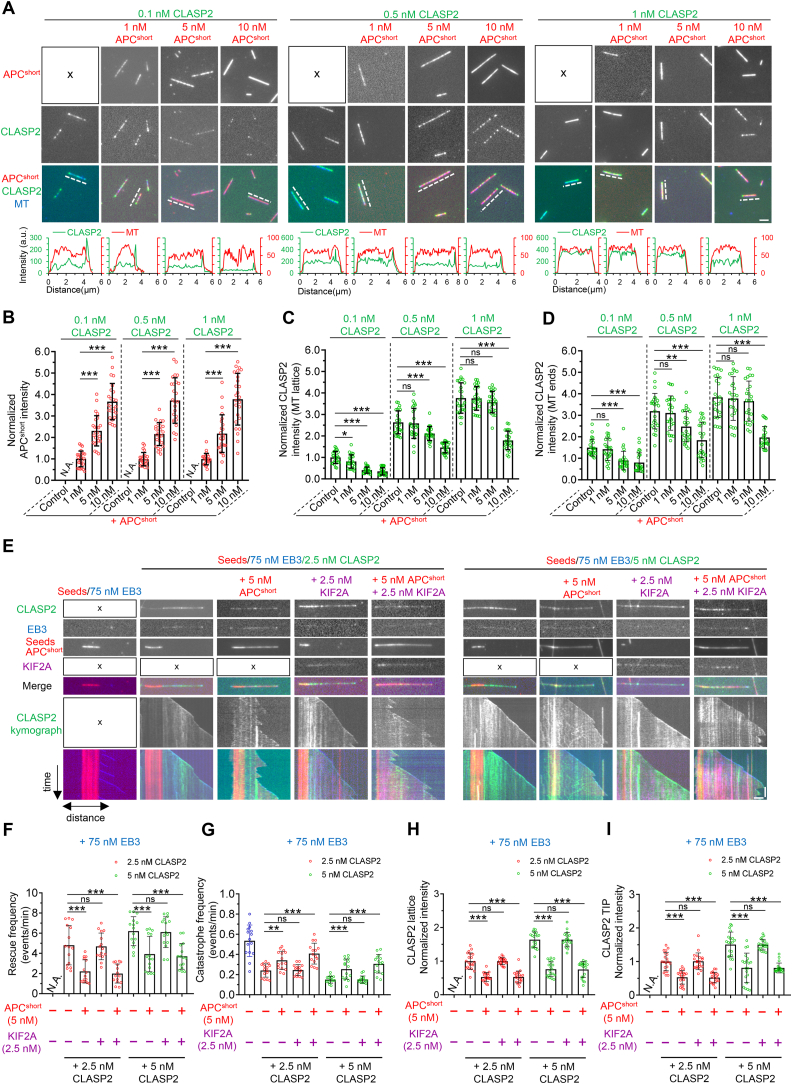
**APC antagonizes the stabilizing effect of CLASP2 *in vitro*** (A) Upper, representative images of GMPCPP-stabilized MTs incubated with 0.1 nM, 0.5 nM and 1 nM GFP-CLASP2 in the absence or presence of the indicated concentrations of mCherry-APC^short^. Lower, the line-scan intensity profiles of CLASP2 and MT as indicated by the dotted lines in the upper images. (B–D) Quantification of the intensities of APC^short^ (B) and CLASP2 along the MT lattices and at MT ends (C and D) for the experiments shown in (A). *n* = 25 MTs from 3 independent experiments. (E) Images and corresponding kymographs of dynamic MTs grown in the presence of 75 nM TagBFP-EB3 alone (left), 75 nM TagBFP-EB3 together with 2.5 nM GFP-CLASP2 (middle) or 5 nM GFP-CLASP2 (right), as well as either 5 nM mCherry- APC^short^ or 2.5 nM KIF2A-SNAP (shown here in purple), or all four proteins together. (F–I) Quantification of rescue frequency (F), catastrophe frequency (G), and the intensities of CLASP2 along the MT lattices (H) and at MT plus ends (I) for the experiments shown in (E). *n* = 15–20 MTs from 3 independent experiments. Data information: Data represent mean ± SD. ∗*p* < 0.05; ∗∗*p* < 0.01; ∗∗∗*p* < 0.001; two-tailed *t*-test (unpaired). Scale bars: horizontal, 2 μm; vertical, 2 min.

Since APC inhibited the localization of CLASPs in cells, we then investigated whether competition between APC and CLASPs exists *in vitro*. We first used GMPCPP-stabilized MTs (seeds) to study the behaviors of these two proteins at different concentrations, tagging APC^short^ and CLASP2 with mCherry and GFP, respectively. Consistent with two recent studies showing that CLASPs can bind the plus-ends of GMPCPP or taxol-stabilized MTs in the absence of GTP (Lawrence et al., 2023; Luo et al., 2023), even at a concentration as low as 0.1 nM, CLASP2 showed strong binding along MT lattices and displayed a preference for one end assumed to be the plus end of the seeds (Fig. 5A). The addition of 1 nM APC^short^ had little impact on the binding behavior of CLASP2 on seeds (Fig. 5A,C and D). However, further increasing the concentration of APC^short^ from 5 nM to 10 nM caused a gradual increase in APC^short^ binding along seeds and a concomitant reduction in the intensities of CLASP2 both along MT lattices and at plus ends (Fig. 5A–D). When CLASP2 concentration was increased to 0.5–1 nM, a relatively high concentration of APC^short^ (10 nM) was required to dramatically reduce the intensities of CLASP2 along MT lattices and at plus ends (Fig. 5A,C and D). Conversely, CLASP2 had little impact on the seeds-binding of APC^short^ at all tested concentrations (Fig. 5A and B). Collectively, these data suggest that APC antagonizes the MT-binding activity of CLASP2.

Next, we examined the combinatorial action of APC, KIF2A, and CLASP2 on dynamic MTs. To address this, we performed *in vitro* dynamics assays in the background of 75 nM EB3. Under such conditions, the addition of 5 nM APC^short^ but not 2.5 nM KIF2A dramatically reduced the binding of CLASP2 (2.5 nM and 5 nM) to the dynamic MT lattices and the growing plus ends. Consistent with previous results (Aher et al., 2018; Lawrence et al., 2018; Majumdar et al., 2018), we observed a CLASP2-dependent increase in MT rescues and a decrease in MT catastrophes at two concentrations, with the effect being more evident at 5 nM than at 2.5 nM (Fig. 5E–I). When added together with 5 nM APC^short^, the rescue frequency decreased by ∼54% and ∼37%, and the catastrophe frequency increased by ∼42% and ∼67% compared to 2.5 nM and 5 nM CLASP2 alone, respectively (Fig. 5E–I). By contrast, the addition of KIF2A by itself had little impact on these parameters but could slightly potentiate the effects of APC^short^ (Fig. 5E–I). These data suggest that APC can antagonize the stabilizing effects of CLASP2 *in vitro*, while KIF2A plays a minor role in this process by assisting APC.

## 3 Discussion

**Fig. 6:**
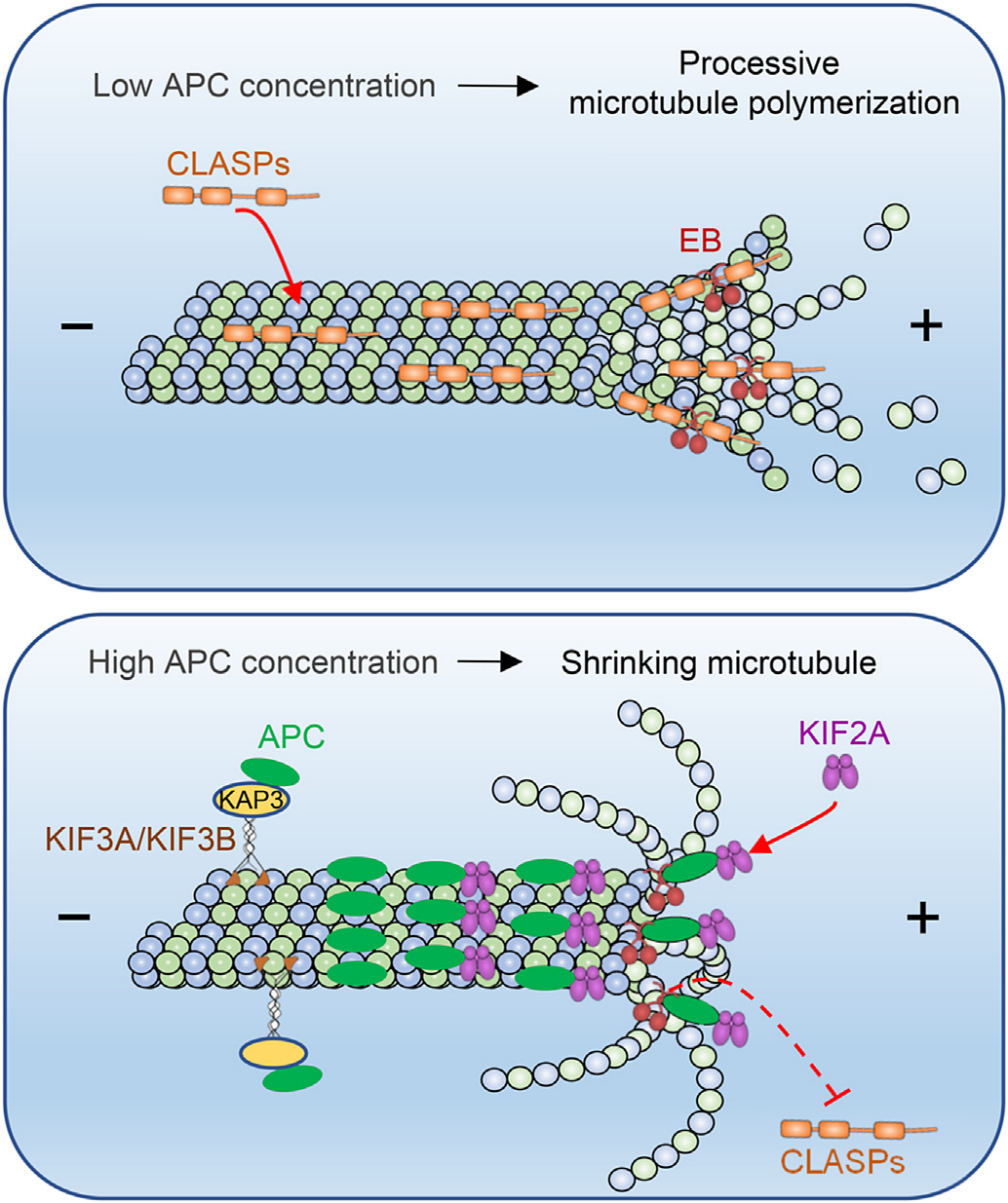
**Model for how APC regulates MT plus-end dynamics** CLASPs stabilize MT plus ends and promote processive MT polymerization in cell regions with low APC concentration or activity. However, APC can be locally concentrated in regions such as extending membranes due to Kinesin-2 complex-mediated transportation and EBs-mediated recruitment. In such regions, APC dissociates MT-stabilizer CLASPs from plus ends and recruits MT-destabilizer KIF2A there, leading to MT depolymerization.

In this study, by combining cellular and *in vitro* reconstitution experiments, we uncover the underlying mechanisms by which APC regulates MT dynamics. APC promotes the destabilizing effect of KIF2A and antagonizes the stabilizing effect of CLASPs, thereby contributing to establishing a dynamic MT network (Fig. 6).

Previous cellular and *in vitro* studies generally suggested APC's role in MT stabilization (Kroboth et al., 2007; Lavrsen et al., 2023; Marshall et al., 2011; Nakamura et al., 2001; Reilein & Nelson, 2005; Togo, 2009; Wen et al., 2004; Yokota et al., 2009; Zumbrunn et al., 2001). However, our current study demonstrates that APC does not affect MT dynamics by itself or in the presence of EBs, which agrees with a recent *in vitro* study (Baumann et al., 2022). The MT stabilization function of APC reported in other cell lines or cultured neurons might be attributed to its interaction with other binding partners.

Since APC2, the homolog of APC promotes MT dynamics in dendrites (Kahn et al., 2018), it is tempting to speculate that APC2 may adopt a mechanism similar to that of APC for its MT destabilizing function. In fact, APC2 also contains two TxI motifs and one TW motif (Figs. S3H–I); therefore, an interesting question to address in the future is whether APC2 can also interact with KIF2A and promote KIF2A-induced catastrophes in the future. If the answer is yes, it is desirable to determine further whether APC2 also outcompetes CLASPs at MT plus ends.

Unlike our previous study showing that KIF2A displays weak MT lattice binding in HeLa cells (Guan et al., 2023), here we found that KIF2A can track MT plus-ends in LLC-PK1 cells through its interaction with APC. These results suggest that the localization and function of KIF2A may be cellular context-dependent, which may be attributed to distinct binding partners or post-translational modifications in different cell lines. It will be interesting to investigate the MT-binding behavior of KIF2A and the contribution of the KIF2A-APC complex in neurons under physiological conditions, as genetic studies in humans and mice have demonstrated the critical roles of KIF2A in brain development (Homma et al., 2003; Poirier et al., 2013; Ruiz-Reig et al., 2024).

Bioinformatics analysis indicates that the N-terminus of KIF2A, which is necessary for APC binding, folds into a Tudor-like domain. Our previous study has shown that KIF2A can also be recruited by CEP170B via this Tudor-like domain to MT minus ends, where they work together to antagonize the major minus-end stabilizer CAMSAPs (Guan et al., 2023). These results demonstrate that the Tudor-like domain is utilized to target the recruitment of KIF2A to both plus and minus ends.

It remains unclear how the competition between APC and CLASPs occurs. In our *in vitro* assay using GMPCPP-stabilized MT seeds, APC decorates the whole MT lattice without enriching at plus ends. However, the presence of APC can reduce the intensity of CLASPs both along MT lattices and at MT plus ends, suggesting that APC outcompetes the overall MT binding of CLASPs. Since low-resolution cryo-EM structure reveals that APC binds along the MT protofilament crest (Serre et al., 2023), it is tempting to speculate that APC might sterically hinder the binding of CLASPs along the MT lattice or at MT ends (Aher et al., 2018; Leano & Slep, 2019; Maki et al., 2015). Alternatively, it has been reported that rescue events often occur shortly after CLASP tracks the shrinking MT end (Aher et al., 2018). Since APC can also track the shrinking end of dynamic MTs (this study; Baumann et al., 2022; Kita et al., 2006), it may interfere with the binding of CLASPs there, thus preventing CLASPs-induced rescues. In cells, APC likely antagonizes CLASPs more effectively than *in vitro*, as it can either be transported by the Kinesin-2 complex or be recruited by EBs to accumulate at MT plus ends (Fig. 6). In addition, since both APC (Breitsprecher et al., 2012; Moseley et al., 2007) and CLASPs (Engel et al., 2014; Rodgers et al., 2023; Tsvetkov et al., 2007) have been reported to bind F-actin, whether the competition between these two proteins on MTs also exist on F-actin awaits further investigation.

In the future, it will be interesting to investigate the interplay between APC, KIF2A, and CLASPs under more physiologically and pathologically relevant conditions. Such studies will enhance our understanding of the diseases caused by mutations in these proteins and may lead to the development of effective medical interventions.

## 4 Materials and methods

### 4.1 DNA constructs and siRNAs

Human APC (NM_000038.6) was purchased from Addgene (#16507). Human EB3 (NM_012326.4), CLASP2α (NM_001365627.1), KIF2A (NM_004520.5), and Paxillin (NM_001080855.3) were gifts from Anna Akhmanova (Utrecht University, the Netherlands).

Vectors used for cloning in this study were as follows: The mCherry-C1 and EGFP-C1 vectors were purchased from Clonetech. The biotinylation tag sequence (MASGLNDIFEAQKIEWHEGGG) was inserted at the N terminus of EGFP in the EGFP-C1 vector to generate the Bio-GFP-C1 vector. The Bio-C1 vector was further modified from the Bio-GFP-C1 vector by removing the coding sequence of the EGFP tag. The pET28a-Strep-GFP-C1 vector was modified from pET28a (Novagen) by replacing the 6xHis tag with the Strep-GFP tag. The pET28a-Strep-mCherry-C1 and pET28a-Strep-TagBFP-C1 vectors were all modified from the pET28a-Strep-GFP-C1 vector by replacing the Strep-GFP tag with Strep-mCherry tag and Strep-TagBFP tag, respectively. The pTT5-Strep-GFP-C1 vector was modified from the pTT5-based pCoofy47 vector purchased from Addgene (#55188) and described previously (Hua & Jiang, 2020). The pTT5-Strep-mCherry-C1, pTT5-Bio-GST-C1, and pTT5-Strep-SNAP-N1 vectors were all modified from the pTT5-Strep-GFP-C1 vector by replacing the Strep-GFP tag with Strep-mCherry tag, Bio-GST tag, and Strep-SNAP tag, respectively.

siRNAs against pig CLASP1 and CLASP2 were purchased from Genepharma. The target sequences were as follows: siRNA CLASP1, 5′-GCCATTATGCCAACTATCT -3′; siRNA CLASP2, 5′-GTTCAGAAAGCCCTCGATG-3′; negative control, 5′-TTCTCCGAACGTGTCACGT-3′.

### 4.2 Cell culture and transfection

All cell lines were cultured in DMEM/F12 (1:1; Biosharp) supplemented with 10% FBS, 100 U/mL penicillin, and 100 μg/mL streptomycin and kept at 37 °C in a 5% CO2 atmosphere. TransIT-LT1 (Mirus) or ExFect Transfection Reagent (Vazyme) were used to transfect plasmids into LLC-PK1 (RRID: CVCL_0391, obtained from China Center for Type Culture Collection) cells for knockout, knock-in, immunofluorescence staining, and live-cell imaging. Polyethylenimine (PEI, Polysciences) was used to transfect plasmids into HEK293T (RRID: CVCL_0063) cells for protein purification and pull-down experiments. siRNAs were transfected with HiPerFect (Qiagen) at 200 nM. All cell lines used in this study were not recently authenticated and tested negative for mycoplasma contamination.

### 4.3 Antibodies

The following antibodies were used for western blotting (WB) and immunofluorescence (IF): rat monoclonal antibodies against APC (Absea, 030903E07, IF-1:100), CLASP1 (Absea, 05008R01A06, IF-1:200), CLASP2 (Absea, 03020R06E03, IF-1:200), α-tubulin YL1/2 (Thermo Fisher Scientific, MA1-80017, IF-1:600); rabbit polyclonal antibodies against GFP (Proteintech, 50430-2-AP, WB-1:2500), detyrosinated α-tubulin (Abcam, ab48389, IF-1:600), KIF2A (Proteintech, 13105-1-AP, IF-1:300, WB-1:1000), paxillin (Proteintech, 10029-1-lg, IF-1:200).

### 4.4 Generation of stable cell lines

The knock-in and knockout cell lines were generated using CRISPR-Cas9 technology.

To tag the APC locus with Strep-GFP at the N terminus, a 2 μg donor construct harboring a blas-T2A-Strep-GFP cassette was cotransfected with 1 μg PX459 bearing single-guide RNA (gRNA: 5′-TATGAAGCTGCAGCCATCCT-3′) into LLC-PK1 cells grown on six-well plates. 24-h post-transfection, cells were first selected with 8 μg/mL puromycin for 2 days and then selected with 5 μg/mL blasticidin for 1 week, followed by serial dilution in 96-well plates. Positive clones were screened by immunofluorescence. The KIF2A-GFP-Strep (gRNA: 5′-AATGCTGATTTAAAGGGCAC-3′) knock-in LLC-PK1 cell line was generated similarly.

To generate the EB3-GFP knock-in LLC-PK1 cell line, we used a method based on random plasmid integration. EB3 was cloned into the GFP-T2A-puro-N1 vector. 2 μg of the resulting construct was then transfected into LLC-PK1 cells grown on six-well plates. 72-h post-transfection, cells were selected with 8 μg/mL puromycin for 2 days, 4–5 days after the removal of puromycin, followed by serial dilution in 96-well plates. Positive clones were screened by immunofluorescence.

To generate APC knockout LLC-PK1 cell line, cells grown on six-well plates were co-transfected with 1 μg PX459 bearing single-guide RNA (5′-TCTTACAAAACTGGAAAC-3′) and 1 μg PX459 bearing single-guide RNA (5′-GCTCTTTTTAAACAGACGTC-3′) targeted with N terminus and C terminus respectively, and then selected with 8 μg/mL puromycin for 2 days. 4–5 days after the removal of puromycin, cells were diluted in 96-well plates to isolate single-cell colonies. Positive clones were screened by immunofluorescence. To generate KIF2A knockout LLC-PK1 cell line, cells grown on six-well plates were transfected with 2 μg PX459 bearing single-guide RNA (5′-TCCAGATGAGGTGATGGCAA-3′), the remaining steps are the same as above.

The following double-engineered cell lines were generated by performing indicated knock-in using the corresponding knockout cell lines as the parental cell lines: APC KO/KIF2A-GFP-Strep knock-in LLC-PK1 cell line; KIF2A KO/Strep-GFP-APC knock-in LLC-PK1 cell line; APC KO/EB3-GFP knock-in LLC-PK1 cell line; KIF2A KO/EB3-GFP knock-in LLC-PK1 cell line.

For genotyping of Strep-GFP-APC knock-in LLC-PK1 cell line, 5′ homology arm-forward primers and 3′ homology arm-reverse primers (APC: 5′-ATGACTGCCTTTTTAATGAGGGTTT-3′ and 5′-AGGACTCTTCCCACTAGGGC-3′) were used.

For genotyping of KIF2A-GFP-Strep knock-in LLC-PK1 cell line, 5′ homology arm-forward primers and 3′ homology arm-reverse primers (KIF2A: 5′-ACACAACAGGATCAGTGGCAT-3′ and 5′-CAGCAGCTATGTCCACTGTCA-3′) were used.

For genotyping of APC knockout LLC-PK1 cell line, APC knockout genotyping-forward primer (5′-CCTTGAAAACAATTCTGACCTGATAGGAA-3′) and APC knockout targeting sequence reverse primer (5′-CCCAGCCTCAGTACAGTGAT-3′) were used.

For genotyping of KIF2A knockout LLC-PK1 cell line, KIF2A knockout genotyping-forward primer (5′-AAACCTCGCTTGGAGGCC-3′) and APC knockout targeting sequence reverse primer (5′-CCGGAAAATGAAGAGGCCGA-3′) were used.

### 4.5 Immunofluorescence

For immunofluorescence, cells were cultured on coverslips in 24-well plates and fixed with different methods. For staining of APC as well as for co-staining of α-tubulin with APC, cells were fixed with 4% formaldehyde in PBS for 5 min at room temperature. For co-staining of CLASP1, CLASP2, and α-tubulin, cells were fixed with −20 °C methanol for 5 min. For staining of detyrosinated α-tubulin, cells were fixed with −20 °C methanol for 5 min and postfixed with 4% formaldehyde in PBS for 5 min at room temperature. Then, cells were permeabilized with 0.1% Triton X-100 in PBS. The subsequent washing and antibody labeling steps were carried out in PBS supplemented with 2% bovine serum albumin (BSA) and 0.05% Tween-20. Finally, coverslips were sequentially rinsed with 70% and 100% ethanol, air-dried, and mounted on glass slides with Vectashield mounting medium (Vector Laboratories). Slides were stored at −20 °C.

Images were captured using Nikon Ni-U with 60 × 1.40 NA oil objective equipped with DS-Qi2 camera (Nikon) for single-slice acquisition.

### 4.6 Wound healing assay

For wound healing assays, cells were seeded directly on the 24-well plate, containing DMEM/F12 (1:1; Biosharp) supplemented with 10% FBS, 100 U/mL penicillin, and 100 μg/mL streptomycin and kept at 37 °C in 5% CO2 atmosphere. Once cells were confluent, the medium was removed and replaced with fresh medium but without FBS for 12 h. After that time, axial wounds were performed using a standard pipette tip. Next, serum-free medium was removed, and cells were washed twice with PBS. Then, cells were replenished with DMEM/F12 medium containing 10% FBS. Cells seeded on the 24-well plates were imaged using Nikon Plan Fluro ADL × 10 objective equipped with HY-500D camera (Hayear).

### 4.7 Protein expression and purification from HEK293T cells

For protein overexpression in HEK293T cells, pTT5-Strep-EGFP-C1 vector was used for APC^short^ and CLASP2, pTT5-Strep-mCherry-C1 vector was used for APC^short^, pTT5-SNAP-Strep-N1 vector was used for KIF2A.

To purify Strep-GFP-tagged APC^short^ and CLASP2, Strep-mCherry-tagged APC^short^, HEK293T cells seeded in two 15-cm dishes were transfected with 20–30 μg DNA per dish using PEI. Cells were treated with 200 ng/mL nocodazole overnight before harvesting. 36-h post-transfection, the medium was removed from dishes, and cells were quickly collected with cold PBS (10 mL for each 15-cm dish) into 15-mL tubes. Then, cells were centrifuged at 1000 × rpm, 4 °C for 5 min to remove the supernatant, and the total pellets were lysed in 2 mL lysis buffer (50 mM HEPES, 300 mM NaCl, and 0.5% Triton X-100, pH 7.4) supplemented with protease inhibitors (Roche) for 10 min on ice. The cell lysate was centrifuged at 14,000 × rpm, 4 °C for 20 min, and the supernatant was collected and incubated with 60–100 μL StrepTactin beads (GE Healthcare) at 4 °C for 45 min. After centrifuging at 3000 × rpm, 4 °C for 1 min, the supernatant was removed and the beads were washed four times with 1 mL lysis buffer and twice with 1 mL wash buffer A (50 mM HEPES, 150 mM NaCl, and 0.01% Triton X, pH 7.4). Finally, proteins were eluted with 60–100 μL elution buffer (50 mM HEPES, 150 mM NaCl, 0.01% Triton X-100, and 2.5 mM desthiobiotin, pH 7.4).

To purify KIF2A-SNAP-Strep, cells were collected 36-h post-transfection without nocodazole treatment and then lysed as described previously. After incubation with the supernatant, beads were washed four times with high-salt wash buffer (50 mM HEPES, 1 M NaCl, and 0.5% Triton X, pH 7.4) and twice with low-salt wash buffer A. Subsequently, for SNAP-tag labeling, beads were incubated with 100 μL wash buffer A supplemented with 10 μM SNAP-Surface Alexa Fluor 647 (NEB) at 4 °C for 1 h in darkness, followed by washing four times with lysis buffer and twice with wash buffer B (50 mM HEPES, 300 mM NaCl, and 0.01% Triton X, pH 7.4). Finally, proteins were eluted with 60 μL elution buffer containing 300 mM instead of 150 mM NaCl.

All purified proteins in this study were snap-frozen in liquid nitrogen, stored at −80 °C, and analyzed by SDS–PAGE.

### 4.8 Protein expression and purification from *E. coli*

EB3 was cloned into a modified pET28a vector (Novagen) containing a Strep-mCherry tag and a Strep-TagBFP tag. The resulting plasmids Strep-mCherry-EB3 and Strep-TagBFP-EB3 were expressed in *E. coli* strain BL21(DE3) and purified with StrepTactin beads.

### 4.9 Pull-down assay

For streptavidin pull-down assays, HEK293T cells seeded in six-well plates were co-transfected with 1 μg Bio-GFP tagged or Bio-tagged bait construct, 1 μg GFP-tagged prey construct and 1 μg BirA construct using PEI. 36-h post-transfection, cells were collected with cold PBS, pelleted, and then lysed in 100 μL lysis buffer (50 mM HEPES, 150 mM NaCl, and 0.5% Triton X-100, pH 7.4) supplemented with protease inhibitors for 5 min on ice. The cell lysate was centrifuged at 14,000 × rpm, 4 °C for 15 min to collect the supernatant. Streptavidin-coated magnetic beads (15 μL for each sample; Streptavidin MagPoly Beads; Smart-lifesciences) were washed twice with lysis buffer. The supernatant was then incubated with Dynabeads at 4 °C for 45 min. Subsequently, beads were washed four times with wash buffer (50 mM HEPES, 150 mM NaCl, and 0.1% Triton X-100, pH 7.4) and then subjected to western blotting analysis.

For GST pull-down assays, HEK293T cells seeded in six-well plates were co-transfected with 1 μg Bio-GST-tagged bait construct, 1 μg GFP-tagged prey construct and 1 μg BirA construct using PEI. Subsequent steps were similar to the procedure described above for streptavidin pulldown assays, except that Glutathione Sepharose beads were used instead of Dynabeads, for GST pull-down assays.

### 4.10 *In vitro* MT dynamics assay

Double-cycled GMPCPP MT seeds were made as described before (Guan et al., 2023). In brief, 8.25 μL tubulin reaction mixture in MRB80 buffer (80 mM PIPES, 4 mM MgCl_2_, and 1 mM EGTA, pH 6.8), which contained 14 μM unlabeled porcine brain tubulin (Cytoskeleton), 2.4 μM rhodamine-tubulin (Cytoskeleton), 3.6 μM biotin-tubulin (Cytoskeleton), and 1 mM GMPCPP (Jena Biosciences), was incubated at 37 °C for 30 min. Subsequently, MTs were pelleted by centrifugation in an Airfuge (Beckman) at ∼28 psi (90,000 rpm, 160,000 g) for 5 min. After carefully removing the supernatant, the pellet was resuspended with 6 μL MRB80 buffer and depolymerized for 20 min on ice. Then, 1 mM fresh GMPCPP was added and a second round of polymerization was performed at 37 °C for 30 min. MT seeds were pelleted as described previously, resuspended in 60 μL MRB80 buffer supplemented with 10% glycerol, snap-frozen in liquid nitrogen, and stored at −80 °C.

*In vitro* MT dynamics assays were performed following the procedure previously described (Jiang et al., 2017). Flow chambers for assays were made by sticking plasma-cleaned glass coverslips onto microscope slides with double-sided tape. The flow chambers were sequentially incubated with 0.2 mg/mL PLL-PEG-biotin (Susos AG) and 1 mg/mL neutravidin (Invitrogen) in MRB80 buffer. Subsequently, GMPCPP seeds were attached to the coverslip by biotin-neutravidin and then blocked with 1 mg/mL κ-casein. The reaction mixture, which consisted of purified protein and MRB80 buffer supplemented with 20 μM porcine brain tubulin, 0.5 μM rhodamine-tubulin, 50 mM KCl, 1 mM GTP, 0.2 mg/mL κ-casein, 0.1% methylcellulose, and oxygen scavenger mix (50 mM glucose, 400 μg/mL glucose oxidase, 200 μg/mL catalase and 4 mM DTT), was added to the flow chamber after centrifugation in an Airfuge at ∼28 psi for 5 min. The flow chambers were sealed with vacuum grease and imaged immediately at 30 °C using a total internal reflection fluorescence (TIRF) microscope. The imaging interval was 3 s unless stated otherwise.

For assays in the presence of purified KIF2A, additional 1 mM ATP was added to the reaction mixture.

For assays with nondynamic MTs, tubulin was excluded from the reaction mixture.

### 4.11 Glass surface and DNA probe preparation

The procedure for glass surface functionalization and Au nanoparticles immobilization were slightly adapted from methods previously developed by the Salaita lab. Circular coverslips (25 mm diameter, 130 μm thickness) were rinsed and sonicated three times in nanopure water (18.2 MΩ cm), and further sonicated in acetone for 20 min. After drying in an oven, the coverslips were activated in the oxygen plasma for 10 min (30 s c.c.m, 300 mttor) to achieve a hydroxylated surface. Subsequently, the coverslips were functionalized with amine groups by incubating the coverslips in ethanol with a 1% v/v (3-Aminopropyl) triethoxysilane for 1 h. The amine-modified coverslips were then rinsed in acetone and dried under a stream of N2. The slides were then annealed for 1 h at 80 °C. The coverslips were then passivated by covering with 200 μL of 0.1 M fresh sodium bicarbonate solution containing 5% w/v mPEG-NHS (MW = 2000) and 0.5% w/v lipoic acid-PEG-NHS (MW = 3400). After overnight incubation at 4 °C, the surface was washed with nanopure water. Subsequently, the 14 nm 5 nm Au solution was added on the coverslip surface to incubate for 30 min at room temperature, followed by three rinses with nanopure water and. The nonspecific bound AuNPs were removed by sonication of the coverslips for 1 min in nanopure water. Finally, 200 nM DNA tension probe in PBS (10 mm sodium phosphate, 1 M NaCl, pH 7.2) was added onto the surface and incubated at room temperature for 1 h. DNA tension probe modified coverslips were rinsed by PBS solution to remove nonspecifically bound probes. These modified coverslips were then assembled into cell imaging chambers and immediately used for cell experiment.

### 4.12 TIRF microscopy

TIRF microscopy was performed on Nikon Eclipse Ti2-E with the perfect focus with the Nikon CFI Apo TIRF 100 × 1.49 NA oil objective, Prime 95B camera (Photometrics), SOLE laser engine (four lasers: 405, 488, 561, and 638 nm; Omicron) and controlled by NIS-Elements software (Nikon). Images were magnified with a 1.5 × intermediate lens on Ti2-E before being projected onto the camera. The resulting pixel size is 73.3 nm/pixel. Stage top incubator INUBG2E-ZILCS (Tokai Hit) was used to keep cells at 37 °C or *in vitro* samples at 30 °C. The imaging medium (DMEM/F12 supplemented with 10% FBS, 100 U/mL penicillin and 100 μg/mL streptomycin) was prewarmed in a water bath at 37 °C.

Optosplit III beamsplitter (Cairn Research Ltd.) was used for simultaneous imaging of green and red fluorescence. Stream acquisition was used for simultaneous imaging of green and red fluorescence in vivo. Sequential acquisition was used for three or four-color imaging experiments.

### 4.13 Image analysis and processing

For measurement of the intensity of indicated protein at the cell periphery, a 20-pixel-wide linear region of interest (ROI) was drawn along the cell edge, in which the intensities of KIF2A (Fig. 1, Fig. 3M), CLASP1/2 (Fig. 2B), MTs (Fig. 2C), integrin force (≥56 pN) (Fig. 2G) and paxillin (Fig. 2 H) at cell periphery were measured.

For measurement of the intensity of indicated protein at the cell edge, a trapezoid region of interest (ROI) was selected to cover the cell edge structure (Fig. 1H).

For measurement of the intensity of indicated protein at the MT plus end, an 8-pixel × 8-pixel square ROI was selected to cover the structure of protein at the MT plus end (Fig. 1F and J).

For measurement of the parameters of plus-end dynamics, the dynamic events of EB3 comets within 4 μm distance from the cell edge were analyzed (Fig. 1L–O).

For measurement of the intensity of detyrosinated-tubulin (Fig. 2E) and GFP-APC (Fig. S3L), a polygon ROI was selected to cover the whole cell.

For measurement of the intensity of KIF2A along GDP-MT (Fig. 4O) and APC alone GMPCPP-MT (Fig. 5B), a 5-pixel-wide linear ROI was selected to cover the microtubule structure.

For the plot of the intensity distribution of CLASP2 alone GMPCPP-MT, a 1-pixel-wide linear ROI was selected to cover the microtubule structure to obtain the intensity profile of indicated proteins (Fig. 5A).

For measurement of the intensity of CLASP2 at the MT lattice and MT end, a 5-pixel-wide linear ROI was selected to cover the MT structure (Fig. 5C), where the four brightest pixels represent the MT end (Fig. 5D).

Kymograph analysis and various quantifications were performed in ImageJ. Plots were generated using Excel (Microsoft) and GraphPad Prism. Images were prepared for publication using ImageJ and Adobe Photoshop.

### 4.14 Mass spectrometry

To identify APC full length interacting proteins, HEK293T cells were co-transfected with Bio-GFP-tagged APC full length with BirA. 36-h post-transfection, Bio-GFP-tagged bait protein and its associated proteins were pulled down using streptavidin beads (Dynabeads M-280); Invitrogen and run on SDS–PAGE gel for 1 cm. After staining the gel with Coomassie dye G-250, the lane was cut and subjected to mass spectrometry using Orbitrap Fusion Lumos mass spectrometer (Thermo Scientific) and Proteome Discoverer 2.4 software (Thermo Scientific) or UHR-QqTOF mass spectrometer (Bruker) and PEAKS 8.5 software (BSI).

### 4.15 Statistical analysis

Statistical analysis was performed with Excel (Microsoft). P-values were determined by unpaired two-tailed *t*-test: ∗*p* < 0.05; ∗∗*p* < 0.01; ∗∗∗*p* < 0.001. Data distribution was assumed to be normal, but this was not formally tested.

## CRediT authorship contribution statement

**Yong Wang:** Writing – original draft, Visualization, Methodology, Investigation, Conceptualization. **Xinping Liu:** Writing – original draft, Visualization, Methodology, Investigation. **Zheng Liu:** Writing – review & editing, Writing – original draft, Visualization, Supervision, Methodology. **Shasha Hua:** Writing – review & editing, Writing – original draft, Visualization, Supervision, Methodology, Conceptualization. **Kai Jiang:** Writing – review & editing, Writing – original draft, Visualization, Supervision, Methodology, Funding acquisition, Conceptualization.

## Conflict of interest

The authors declare no competing financial interests.

## Declaration of competing interest

The authors declare that they have no known competing financial interests or personal relationships that could have appeared to influence the work reported in this paper.
