# Supplementary figures and images for: APC orchestrates microtubule dynamics by acting as a positive regulator of KIF2A and a negative regulator of CLASPs
Source: Cell Insight. 2024 Oct 11;4(1):100210. doi: 10.1016/j.cellin.2024.100210 (PMC11617872; doi:10.1016/j.cellin.2024.100210)

# Fig S1

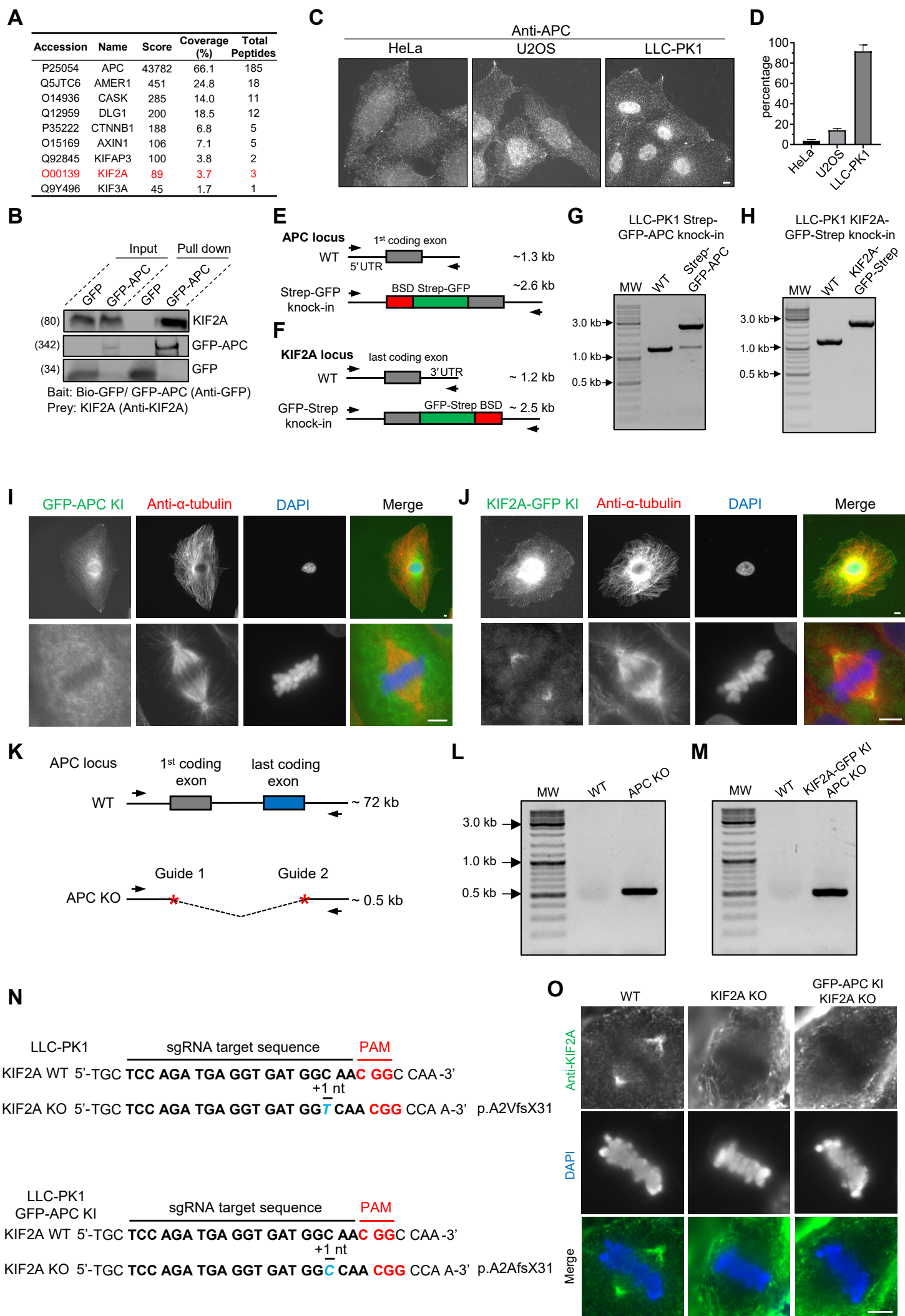

**Fig S2**

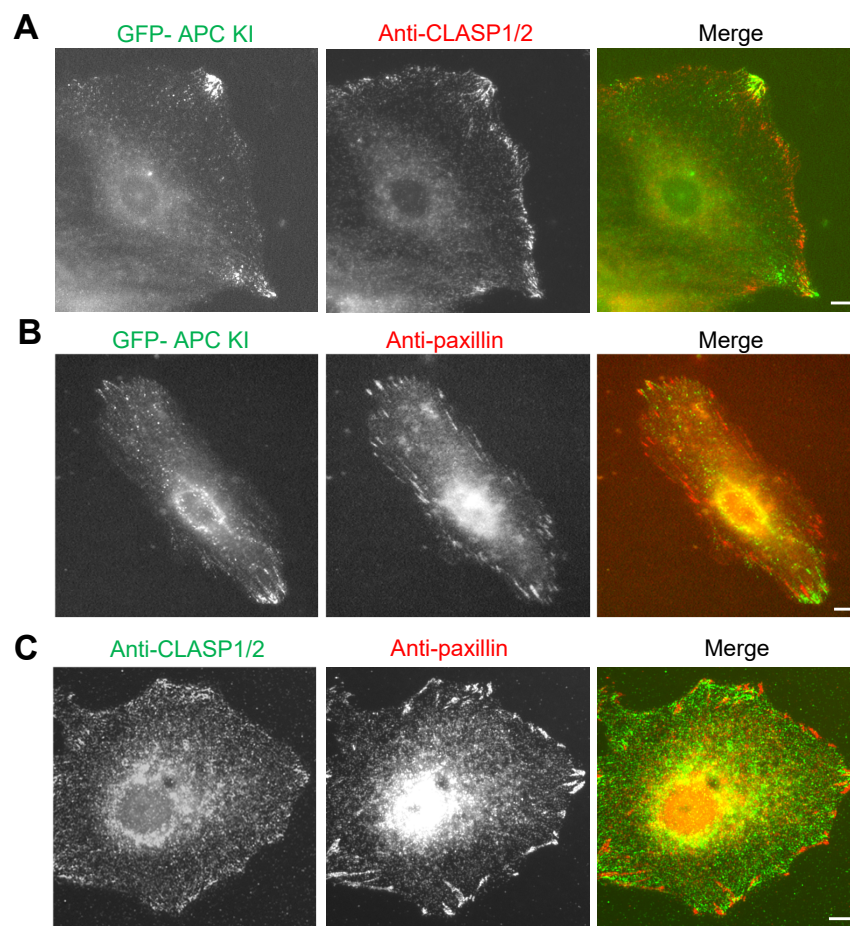



Fig S4

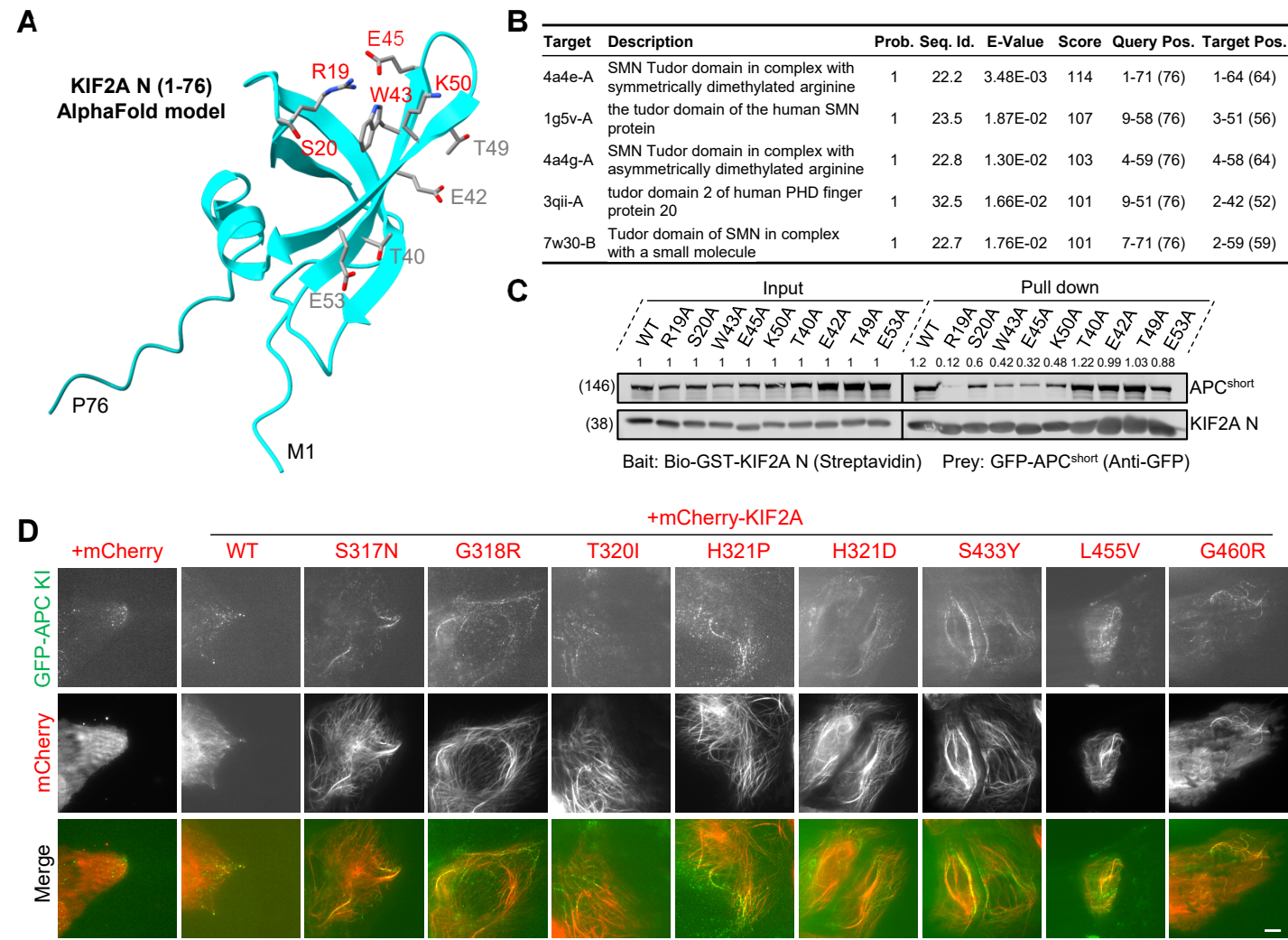

Supplement: Multimedia component 1 [file mmc1.pdf]
