# Supplementary material for: APC orchestrates microtubule dynamics by acting as a positive regulator of KIF2A and a negative regulator of CLASPs
Source: Cell Insight. 2024 Oct 11;4(1):100210. doi: 10.1016/j.cellin.2024.100210 (PMC11617872; doi:10.1016/j.cellin.2024.100210)
Supplement: Multimedia component 2 [file mmc2.docx]

**Figure S1. Characterization of APC and KIF2A knock-in and knockout (KO) LLC-PK1 cell lines**

(A) Mass spectrometry results of streptavidin pull-down from HEK293T cells transiently expressing Bio-GFP-APC.

(B) Streptavidin pull-down assay with extracts of HEK293T cells expressing Bio-GFP vector and Bio-GFP-tagged APC full length, analyzed by western blotting with KIF2A antibody. The Bio-GFP-tagged bait proteins were detected by GFP antibody.

(C) Immunofluorescence staining of APC in HeLa, U2OS, and LLC-PK1 cells.

(D) Quantification of the percentage of cells with prominent APC clustering at the cell edge being observed.

(E-F) Schematic illustration of primer sets and the expected PCR products of Strep-GFP-APC knock-in LLC-PK1 cell line (E) and KIF2A-GFP-Strep knock-in LLC-PK1 cell line (F). BSD, blasticidin S deaminase.

(G-H) PCR genotyping shows that the Strep-GFP-APC knock-in LLC-PK1 cell line is heterozygous (G) and that the KIF2A-GFP-Strep knock-in LLC-PK1 cell line is homozygous (H) in this study.

(I-J) Immunofluorescence staining of α-tubulin and DNA (DAPI) in Strep-GFP-APC knock-in LLC-PK1 cells (I) and KIF2A-GFP-Strep knock-in LLC-PK1 cells (J) in interphase and mitosis.

(K) Schematic illustration of primer sets and the expected PCR products of APC KO LLC-PK1 cell line.

(L-M) PCR genotyping in WT and APC KO LLC-PK1 cell line (L) or APC KO/KIF2A-GFP-Strep knock-in double-engineered LLC-PK1 cell line (M).

(N) Sanger sequencing results of KIF2A KO LLC-PK1 cell line or KIF2A KO/Strep-GFP-APC knock-in double-engineered LLC-PK1 cell line revealed one nucleotide (nt) insertion. One nt insertion will result in p.A2VfsX31 or p.A2AfsX31. sgRNA, single-guide RNA.

(O) Immunofluorescence staining of KIF2A and DAPI in control, KIF2A KO, and KIF2A KO/Strep-GFP-APC knock-in double-engineered LLC-PK1 cell line in mitosis.

Data information: Data represent mean ± SD. Scale bars, 5 µm.

**Figure S2.** **Partial colocalization of APC and CLASP1/2**

(A) Immunofluorescence staining of CLASP1/2 in Strep-GFP-APC knock-in LLC-PK1 cell line.

(B) Immunofluorescence staining of paxillin in Strep-GFP-APC knock-in LLC-PK1 cell line.

(C) Immunofluorescence staining of CLASP1/2 (green) and paxillin (red) in LLC-PK1 cells.

Data information: Scale bar, 5 µm.

**Figure S3.** **Biochemical characterization and mutational analysis of the interaction between APC and KIF2A**

(A) Schematic overview of the domain organization of APC and the deletion mutants. N1: oligomerization domain; M1: Armadillo repeat domain (KAP3 binding) and 15-aa, 20-aa, SAMP repeats (β-catenin and Axin binding); C1: basic domain and EB-binding domain containing multiple SxIP motifs.

(B) Schematic overview of the domain organization of APC C1 and its truncations and summary of their interactions with KIF2A N.

(C) Streptavidin pull-down assay with extracts of HEK293T cells expressing Bio-GST-tagged KIF2A N (bait) together with GFP-tagged APC C1 and its indicated truncations (prey), analyzed by western blotting with GFP antibody. The Bio-GST-tagged bait proteins were detected by IRDye^®^ 680RD Streptavidin.

(D) Schematic overview of the domain organization of APC^short^ and its truncations and summary of their interactions with KIF2A C.

(E) Streptavidin pull-down assays with extracts of HEK293T cells expressing Bio-GFP-tagged KIF2A C (bait) together with the GFP-tagged APC^short^ and its indicated truncations (prey), analyzed by western blotting with GFP antibody.

(F) Schematic overview of the domain organization of APC^short^ and its truncations and summary of their interactions with KIF2A C.

(G) Streptavidin pull-down assays with extracts of HEK293T cells expressing Bio-GFP-tagged KIF2A C (bait) together with the GFP-tagged APC^short^ and its indicated truncations (prey), analyzed by western blotting with GFP antibody.

(H) Alignment of KIF2A N-binding region in APC and APC2 from five vertebrate species. The threonine and tryptophan residues critical for KIF2A N-binding were indicated with asterisks.

(I) Alignment of KIF2A C-binding region in APC and APC2 from five vertebrate species. The threonine, methionine (or phenylalanine), and isoleucine residues critical for KIF2A C-binding were indicated with asterisks.

(J) Streptavidin pull-down assays with extracts of HEK293T cells expressing Bio-GST-tagged KIF2A N (bait) together with indicated GFP-LZ-tagged APC single repeats and corresponding mutants (prey), analyzed by western blotting with GFP antibody. The Bio-GST-tagged bait proteins were detected by IRDye® 680RD Streptavidin. LZ, leucine zipper from GCN4, was used as a dimerization domain.

(K) Streptavidin pull-down assays with extracts of HEK293T cells expressing Bio-GFP-tagged KIF2A C (bait) together with indicated GFP-LZ-tagged APC single repeats and corresponding mutants (prey), analyzed by western blotting with GFP antibody.

(L) Quantification of intensities of GFP-APC in Strep-GFP-APC knock in LLC-PK1 cell line or APC KO cell line transiently transfected with GFP-tagged WT APC or its indicated mutants. n = 25 cells from three independent experiments.

Data information: Data represent mean ± SD. ***p < 0.001; two-tailed t-test (unpaired).

**Figure S4.** **Mutational analysis of critical residues in KIF2A facilitating the interaction with APC**

(A) AlphaFold predicts the N-terminal globular domain of KIF2A (1-76 aa) to be a barrel-like structure. Residues on top of the barrel (marked in red) but not at its side (marked in grey) are critical for APC binding.

(B) Structure similarity search against the PDB database by Foldseek reveals that the folding of KIF2A (1-76 aa) is similar to that of the Tudor domains recognizing methylated lysine or arginine residues.

(C) Streptavidin pull-down assay with extracts of HEK293T cells expressing Bio-GST-tagged KIF2A N or its indicated mutants (bait) together with GFP-tagged APC^short^ (prey), analyzed by western blotting with GFP antibody. The Bio-GST-tagged bait proteins were detected by IRDye^®^ 680RD Streptavidin.

(D) TIRF live-cell images of Strep-GFP-APC knock-in LLC-PK1 cell line transiently transfected with control mCherry vector, mCherry-tagged WT KIF2A, or its indicated mutants.

Data information: Scale bars, 5 µm.
